# Limber Pine (*Pinus flexilis* James), a Flexible Generalist of Forest Communities in the Intermountain West

**DOI:** 10.1371/journal.pone.0160324

**Published:** 2016-08-30

**Authors:** Marcella A. Windmuller-Campione, James N. Long

**Affiliations:** 1 Department of Forest Resources, College of Food, Agriculture and Natural Resource Sciences, University of Minnesota, St. Paul, Minnesota, United States of America; 2 Department of Wildland Resources & Ecology Center, Utah State University, Logan, Utah, United States of America; Natural Resources Canada, CANADA

## Abstract

As forest communities continue to experience interactions between climate change and shifting disturbance regimes, there is an increased need to link ecological understanding to applied management. Limber pine (*Pinus flexilis* James.), an understudied species of western North America, has been documented to dominate harsh environments and thought to be competitively excluded from mesic environments. An observational study was conducted using the Forest Inventory and Analysis Database (FIAD) to test the competitive exclusion hypothesis across a broad elevational and geographic area within the Intermountain West, USA. We anticipated that competitive exclusion would result in limber pine’s absence from mid-elevation forest communities, creating a bi-modal distribution. Using the FIAD database, limber pine was observed to occur with 22 different overstory species, which represents a surprising number of the woody, overstory species commonly observed in the Intermountain West. There were no biologically significant relationships between measures of annual precipitation, annual temperature, or climatic indices (i.e. Ombrothermic Index) and limber pine dominance. Limber pine was observed to be a consistent component of forest communities across elevation classes. Of the plots that contained limber pine regeneration, nearly half did not have a live or dead limber pine in the overstory. However, limber pine regeneration was greater in plots with higher limber pine basal area and higher average annual precipitation. Our results suggest limber pine is an important habitat generalist, playing more than one functional role in forest communities. Generalists, like limber pine, may be increasingly important, as managers are challenged to build resistance and resilience to future conditions in western forests. Additional research is needed to understand how different silvicultural systems can be used to maintain multi-species forest communities.

## Introduction

Historically forest research was primarily focused on commercially productive species or forest communities [1]. Forest communities, however, are increasingly being managed for a broader set of goals and objectives [2]. Nevertheless, there is still relatively limited information on the forest dynamics of non-commercial systems. An example of these systems includes the high elevation, five-needle white pine species (the high five) of the middle latitudes of western North America.

The “high five” includes six species of five-needle white pines, belonging to the Family Pinaceae, Genus *Pinus* and the subgenus *Strobus* [3]. They have been grouped together because of morphological and ecological similarities [4]. Individuals are commonly dominant in harsh environments at treeline throughout western North America and serve as important keystone species [5]. They provide valuable wildlife habitat [6], serve as a wildlife food source [7], [8], influence snow dynamics and the timing of run-off [9], and serve as important symbols of strength and endurance for mountain visitors [10].

One common way to describe forest communities in the Intermountain West is based on dominant overstory species at different elevation zones. Compared to most forest regions in North America, the Intermountain West has limited overstory tree diversity; many of the different forest zones have less than three different overstory species [11]. Common forest zones from lower to upper elevation in Intermountain West are pinyon-juniper, ponderosa pine, Douglas-fir, lodgepole pine, spruce-fir, and high elevation 5-needle pines [4].

The high five commonly occur and dominate the highest forest elevation zone. Limber pine (*Pinus flexilis* James), can occur and even be the dominant species at both upper and lower treeline across many of the mountain ranges of western North America [12]. This distribution, and the associated broad environmental gradient, is presumably reflective of limber pine’s broad fundamental niche or potential habitat. However, limber pine’s realized niche has been described as much smaller due to its poor competitive ability [10], [12], [13]; see [14]-[16] for an alternative. Under moderate environmental conditions in the montane and subalpine forest zone, limber pine can be described as an early seral species. It may be the first species to establish after stand-replacing disturbances but is outcompeted by conifer species like subalpine fir (*Abies lasiocarpa* (Hook.) Nutt.) and Engelmann spruce (*Picea engelmannii* Parry. ex Engelm.) [17]–[19]. This can result in limber pine being a minor component of these spruce-fir forests. It is only on harsh, rocky, xeric sites (centrifugal theory of community organization *sensu* [20]) where limber pine can form climax communities. Similar patterns of establishment and facilitation have been observed between limber pine and Douglas-fir (*Pseudotsuga menziesii* (Mirb.) Franco) at lower elevations [21–23]. Based on this description, limber pine’s functional role could be described as a stress tolerator with some ruderal qualities [24], [25].

Limber pine is being negatively impacted by interactions between mountain pine beetle (*Dendroctonus ponderosae* Hopkins), white pine blister rust (*Cronartium ribicola* J. C. Fisch. ex Rabenh.*)*, and changing climatic conditions [26]. Researchers have observed some levels of resistance to mountain pine beetle and white pine blister [27], [28]. However, a better understanding is needed of the functional role of limber pine in forest communities to aid the management and restoration of this species.

Limber pine has been described as being competitively excluded from more moderate environmental conditions, creating a bi-modal distribution [17], [18], [29–31]. However, this competitive exclusion hypothesis has not been thoroughly examined across limber pine’s broad elevational and geographic distribution. To explore the competitive exclusion hypothesis, data from the Forest Inventory and Analysis Database (FIAD) were used to examine the relationship between limber pine and environmental variables. Our expectation was that limber pine would have a bi-modal distribution with peaks of dominance at higher and lower elevations. Additionally, we expected limber pine dominance would be strongly correlated to environmental variables (temperature, precipitation) and climatic indices. Previous studies on limber pine dynamics have used purposive sampling with a narrowly defined geographic range and/or stand structure [15], [18], [32], [33]. The FIAD is representative of stand conditions across the United States, allowing us to quantify the functional role of limber pine in forest communities across a broad regional and elevational range. This increased understanding will be important as natural resource managers focus on building resistance and resilience to current and future forest threats.

## Methods

### Study area

The Intermountain West encompasses Montana, Idaho, Nevada, Utah, Wyoming, Colorado, New Mexico, and Arizona. Across these eight states, there are many diverse ecosystems including numerous mountain ranges, shrub steppes, and deserts. The major ecoregions that were the focus of this study were the Southern Rocky Mountain Steppe, Middle Rocky Mountain Steppe, Northern Rocky Mountain Forest- Steppe, and the Nevada–Utah Mountain Semidesert [34].

The climate of the Intermountain West is arid (< 250 mm/yr precipitation) to semi-arid (250–500 mm/yr precipitation) with higher elevations receiving more than 1200 mm/yr of annual precipitation due to orographic uplift [35], [36]. The majority of precipitation falls as winter snow but in the southern portions (New Mexico, Arizona, southern Utah, and southern Colorado) the North American Monsoon provides important summer precipitation [37]. Yearly precipitation can be highly variable, resulting in both high and low precipitation years [38]. Additionally, local, small-scale physiographic features (i.e. aspect, elevation, and slope) create high variability in moisture patterns [38], [39].

### Study design

A query of the Forest Inventory and Analysis Database (FIADB) in 2013 located all FIA plots containing limber pine in the overstory and regeneration layer within the Intermountain West. The current FIA sampling design is approximately 0.067 ha and includes four 7.32 m radius subplots. On each subplot, overstory trees greater than 12.7 cm at dbh (diameter at breast height) were measured. Each subplot contains a 13.5 m^2^ circular microplot where saplings, trees between 2.4 cm and 12.7 cm dbh, and seedlings, trees less than 2.4 cm dbh, were measured. Only Phase 2 data were used in analysis. Additional data are collected in Phase 3 plots, including soil attributes, but this collection is done on only a subset of Phase 2 plots (approximately 1/16^th^), greatly reducing our sample size. O’Connell and colleagues [40] provide additional details on the sample design. Some states were in the process of beginning their second round of annual inventories resulting in two years of data. The most recent sampling year was used so there were no repeated measurements within the dataset.

The data were separated by overstory and regenerating trees. Overstory trees were defined as limber pine with a dbh greater than 2.54 cm. Live and dead trees were recorded for all trees ≥ 12.7 cm in dbh; for trees between 2.54 and 12.7 cm only live trees were recorded. Regenerating limber pine trees were any individuals less than 2.54 cm in dbh but greater than 15.24 cm in height and only recorded if alive.

For a plot to be included in the final data set, plots could only have one condition class; forest conditions are defined as distinct changes in vegetation cover or changes in land management boundaries [40]. Multiple condition classes were excluded since determining boundaries between conditions in the field can be difficult and multiple conditions across the subplots increase the complexity of analysis, potentially increasing errors. Additionally, plots needed to be associated with long-term climate data from the PRISM climate database [41]. This resulted in a total of 841 plots with limber pine present in either the overstory or regeneration layer. Of these plots, the majority, 673 plots, had limber pine present in the overstory with only 28% (191 plots) containing limber pine in the regeneration layer. There were an additional 168 plots that only contained regenerating limber pine. This low percentage of plots with limber pine regeneration may be due to sampling design; seedlings are only measured in the microplot. Seedlings may be present in the subplot but are not recorded if they fall outside of the microplot, resulting in fewer plots with limber pine regeneration.

### Statistics

Descriptive statistics of stand, site, and environmental variables were calculated using both the PRISM and FIADB databases. Overstory stand density metrics were expanded to trees per hectare (tph) and basal area per hectare (m^2^ha^-1^); the regeneration layer was also expanded to tph. Two climatic indices were calculated: Aridity Index [42] and the Ombrothermic Index [43]. The Aridity Index (A_m_) is calculated using mean annual temperature and precipitation values.

$$A_{m}=\frac{P}{\left(T+10\right)}$$

P=annual precipitation(cm)

T=annual mean temperature(°C)

The Ombrothermic Index (OI) takes into account length of the growing season by utilizing temperature and precipitation values for months where the average temperatures are above 0°C.

$$OI=(\frac{P_{p}}{T_{p}})\times \;10$$

$${\text{P}}_{\text{p}}=\text{total average precipitation of months where average temperature is greater than}\;0{}^{\circ}\text{C}$$

$${\text{T}}_{\text{p}}=\text{sum of monthly average temperature of months where average temperature is greater than}\;0{}^{\circ}\text{C}$$

Mean OI values for common forest types across the Intermountain West range from values in the 20’s to values around 100 [44]. Our data set captures the range of potential OI values across the Intermountain West. Since sites occurred across a wide latitudinal range (35.2° – 48.9°), an elevation correction (EC) was used. A value of 129.4 m was added for every 1° difference from the minimum latitude [45]. Figures and table detail when elevation values were corrected or uncorrected.

To assess the competitive exclusion hypothesis, linear regression was used to explore the relationships between environmental variables, climate indices, and limber pine density and dominance. To further explore the dataset, additional standardization was done for composition due to the wide range of total plot basal area. Percent composition was the basal area of the individual species divided by the total plot basal area multiplied by 100. Percent limber pine basal area was grouped into three classes: minor (<25% limber pine), moderate (25–75% limber pine), and major (>75% limber pine). Average yearly precipitation was also grouped into three classes: < 400 mm, which is characteristic of the average yearly precipitation for pinyon-juniper woodlands [46]; 400–900 mm, which is characteristic of the average yearly precipitation for mid-elevation forests [47]; and > 900 mm, which is characteristic of average yearly precipitation for spruce-fir forests [48]. Stand age and elevation were also categorized and represent stages of stand develop and broad forest zones, respectively.

To further explore the competitive exclusion hypothesis, a subset of data, plots with average annual precipitation between 400–900 mm and stands ages between 101–250, was used to explore potential differences in limber pine forest dynamics. This subset of data was used since limber pine has been described as being competitively excluded under moderate environmental conditions [17]-[19]. Using 5 cm diameter classes, diameter distributions with average basal area per hectare (m^2^ha^-1^) were created for each limber pine dominance class.

Conditional interference trees with program ctree [49] in the statistical program R were used to explore the relationship between environmental variables (average yearly precipitation, average yearly temperature), stand variables (total overstory basal area, limber pine basal area, percent limber pine), and limber pine regeneration.

## Results

### Limber pine distribution & dominance

**Fig 1:**
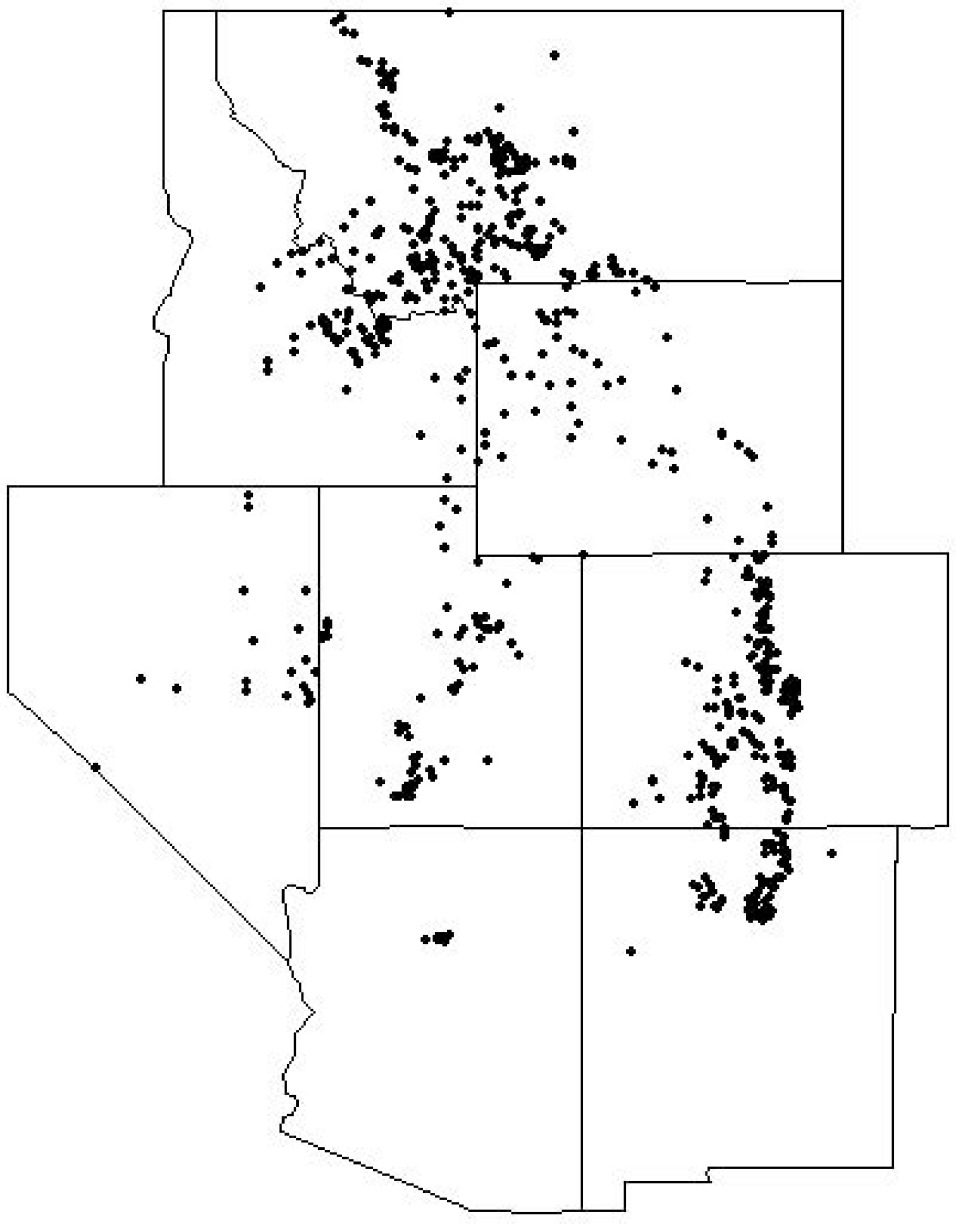
Map of limber pine FIA sampling locations across the Intermountain West.

**Fig 2:**
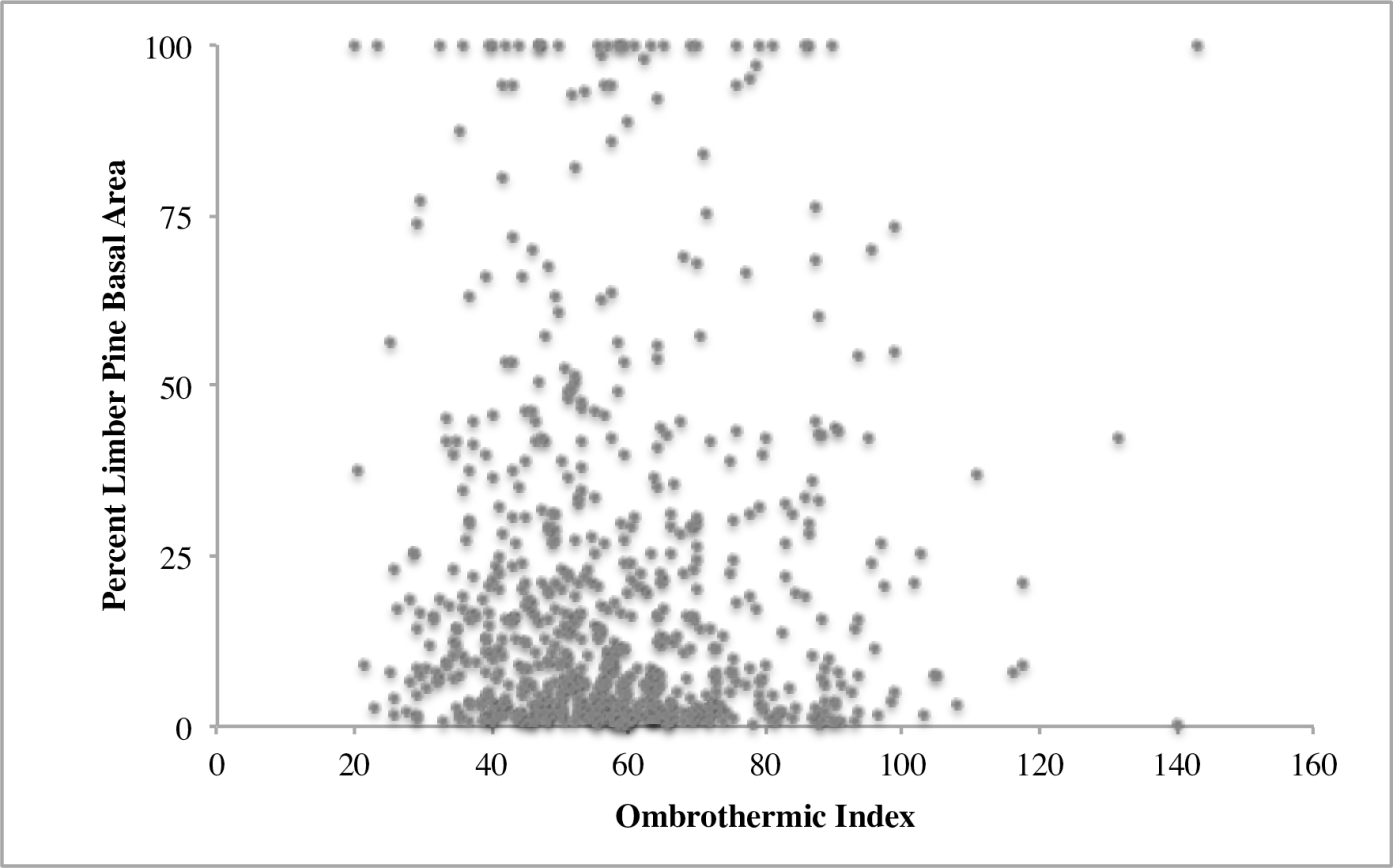
The relationship between limber pine dominance and the Ombrothermic Index (OI). OI represents important growing season conditions with lower values representing conditions that are warmer and drier and higher values representing cooler, moister conditions.

Across the Intermountain West, overstory limber pine was observed across a wide range of environmental conditions and a broad geographic area (Table 1; Fig 1). Limber pine overstory dominance had low correlation (R^2^ < 5%) with the Ombrothermic Index (OI) (Fig 2). Average OI values for common forest types in the Intermountain West range from 20 for pinyon-juniper to 100 for spruce-fir [44]. Low correlations between limber pine overstory dominance and measures of average annual precipitation and temperature and the aridity index were also observed.

**Table 1:** Descriptive statistics for plots containing overstory limber pine (> 2.54 cm dbh) across the Intermountain West.

	Total basal area (m^2^ha^-1^)	Total limber pine basal area (m^2^ha^-1^)	Live limber pine basal area (m^2^ha^-1^)	Dead limber pine basal area (m^2^ha^-1^)	Percent limber pine	Elevation* (m)	Yearly precipitation (mm)	Yearly temperature (C°)
Average	28.3	4.9	3.4	1.4	21.8	2497.9	624.6	4.1
Standard error	0.6	0.3	0.2	0.1	1.0	18.7	7.6	0.1
Minimum	0.2	0.1	0.0	0.0	0.3	1177.4	264.0	-3.0
Maximum	117.7	43.9	39.2	31.9	100.0	3547.0	1767.0	10.0

*Uncorrected elevations were used.

**Fig 3:**
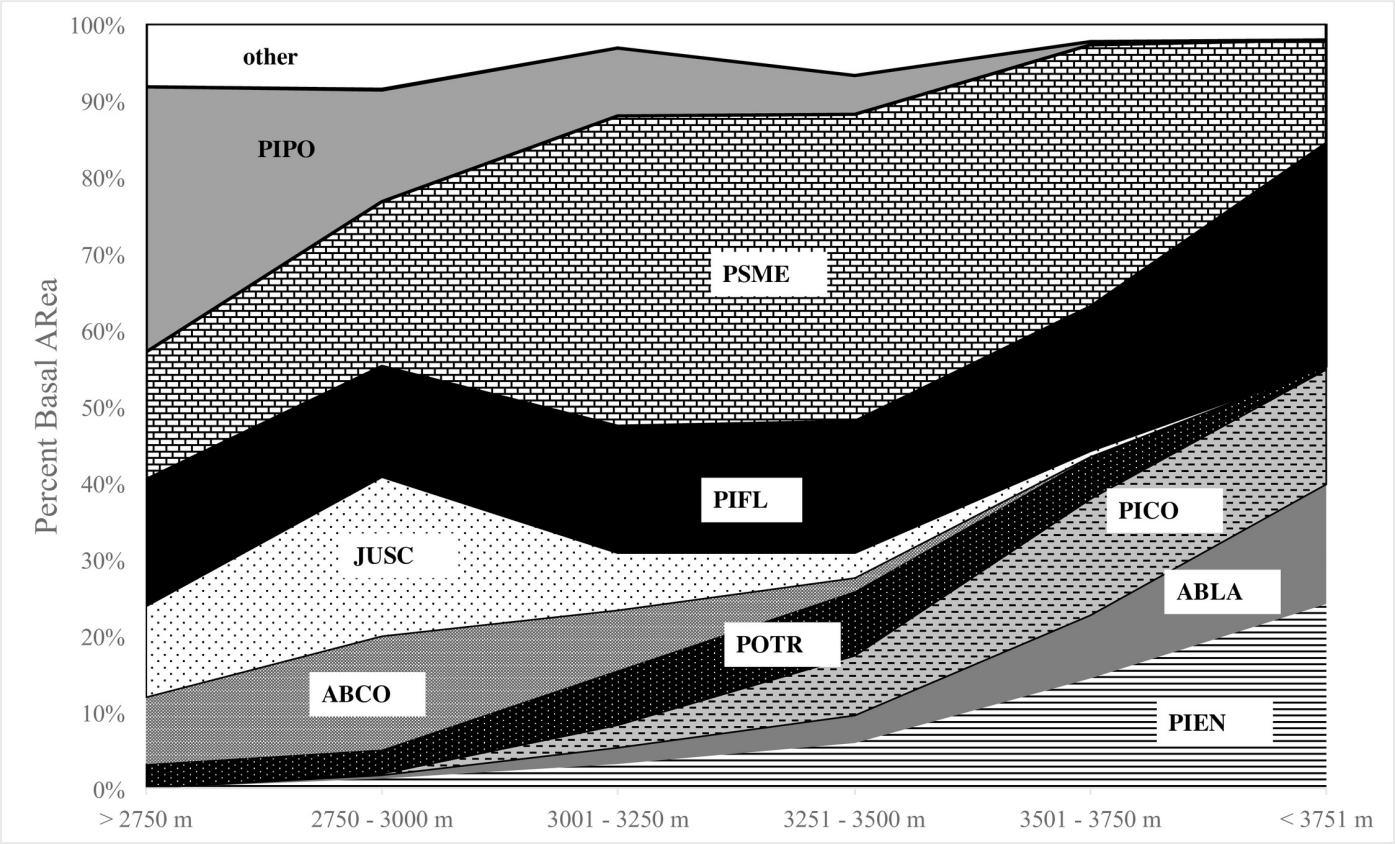
Percent basal area of the most abundant species by elevation class. Latitudinally corrected elevation (EC) was used. Species are coded based on the first two letters of the genus and species. A total of 22 unique overstory species were observed with the other category represents species with less than <5% basal area by elevation class. Percent basal area includes live overstory (>2.54 cm dbh) trees. Species codes: PIPO = *Pinus ponderosa*; PSME = *Pseudotsuga menziesii*; PIFL = *Pinus flexilis*; JUSC = *Juniperus scopulorum*; ABCO = *Abies concolor*; POTR = *Populus tremuloides*; PIEN = Picea engelmannii; ABLA = *Abies lasiocarpa*; PICO = *Pinus contorta*.

Limber pine was observed to occur with twenty-two different overstory species and was observed, on average, to be a consistent component in the overstory across broad elevational classes when present on FIA plots (Fig 3). Many tree species, especially those typically restricted to lower or upper elevations (i.e. Rocky Mountain juniper (*Juniperus scopulorum* Sarg.) and Engelmann spruce), were not present in all the elevation classes. Only a few species, including limber pine, Douglas-fir, and aspen (*Populus tremuloides* Michx.) occurred across all elevation classes in stands that contained limber pine. Douglas-fir displayed a uni-modal distribution, with dominance peaking in the mid-elevation classes and decreasing in both lower and upper elevation classes when co-occurring with limber pine. However, limber pine dominance displayed neither a uni-modal nor bi-modal distribution. Dominance, measured as percent of stand basal, ranged from 14–19% in all elevation classes except for the highest class (> 3751 m) where it was 29%. Aspen also displayed a relatively consistent distribution of ~5% of the basal area in stands with limber pine.

### Diameter distributions

A subset of the data was used to further explore the competitive exclusion hypothesis; this subset includes plots with a stand age between 101–250 years that received between 400 and 900 mm of annual precipitation. This subset represents approximately 42% (286 plots) of the data. The majority of this subset of data (219) had limber pine as a minor component of the stand, composing less than 25% of the overstory basal area. Limber pine was a major component, greater than 75% of the overstory basal area, in 10 plots; 57 plots had limber pine as a moderate component (25–75%).

**Fig 4:**
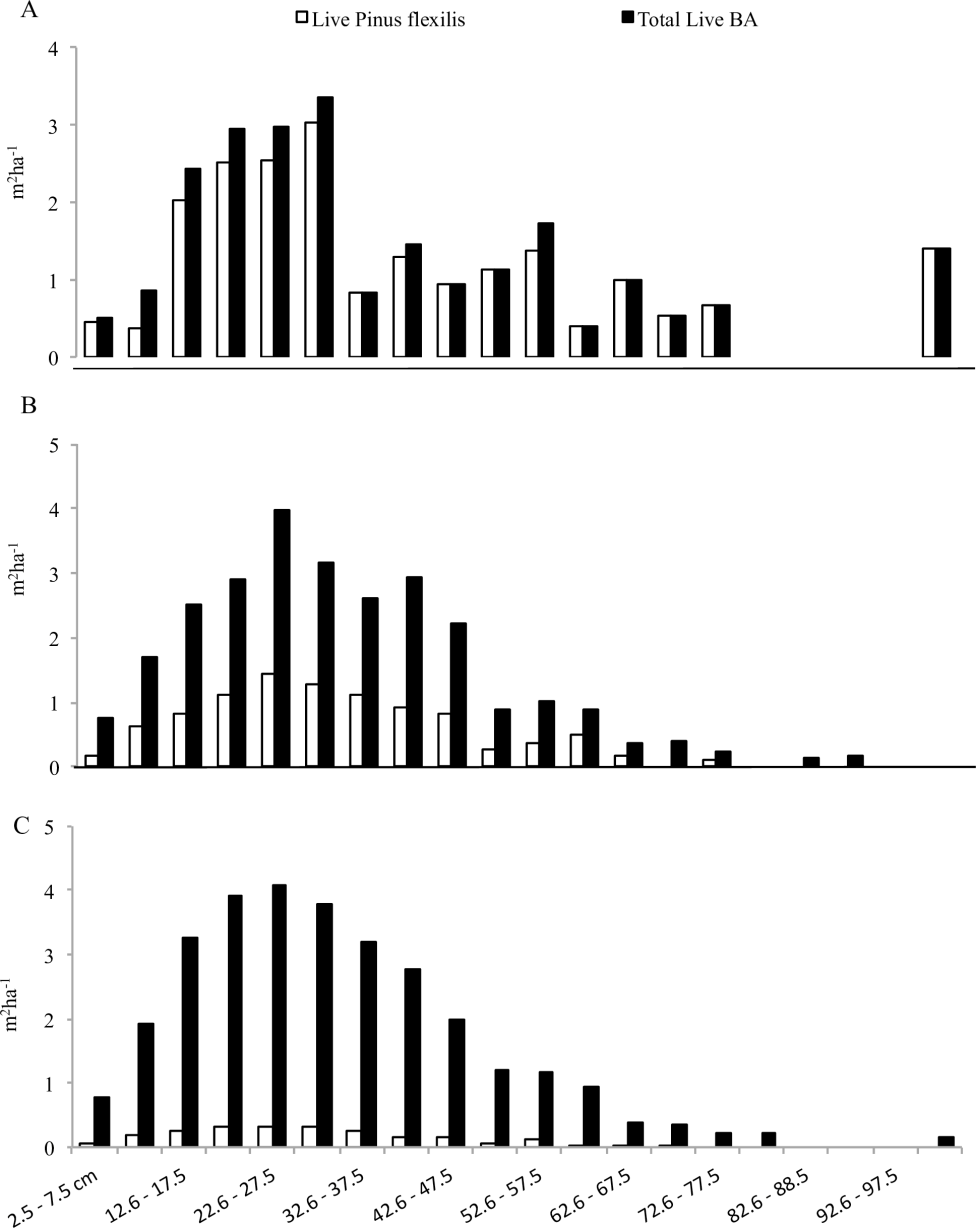
Diameter distributions for limber pine plots with a stand age between 101–250 years and average yearly precipitation between 400–900 mm broken up by average percent limber pine. A) Minor limber pine composition (<25% of the basal area); B) Moderate percent limber pine composition (25–75% of the basal area); and C) Major percent limber pine (>75% of the basal area).

Total live basal area for all species was remarkably similar between stands with minor, moderate, and major limber pine dominance (Fig 4). In all three instances, limber pine was observed across the majority of the diameter classes. Additionally, all three dominance classes had limber pine in the smallest diameter classes (<12.6 cm dbh), reflecting recruitment of limber pine regeneration.

### Limber pine regeneration

Limber pine regeneration was observed under similar environmental conditions as overstory limber pines. Across all plots with limber pine regeneration, average density was 645 tph with a standard error of 62 tph. The maximum regeneration density was 16,850 tph; this plot was excluded from future analysis. There were weak but significant positive relationships between limber pine regeneration density and total yearly precipitation (p<0.001) and live limber pine basal area (p = 0.02). There was no relationship between average July precipitation, average July temperature, or yearly average temperature with limber pine regeneration.

**Fig 5:**
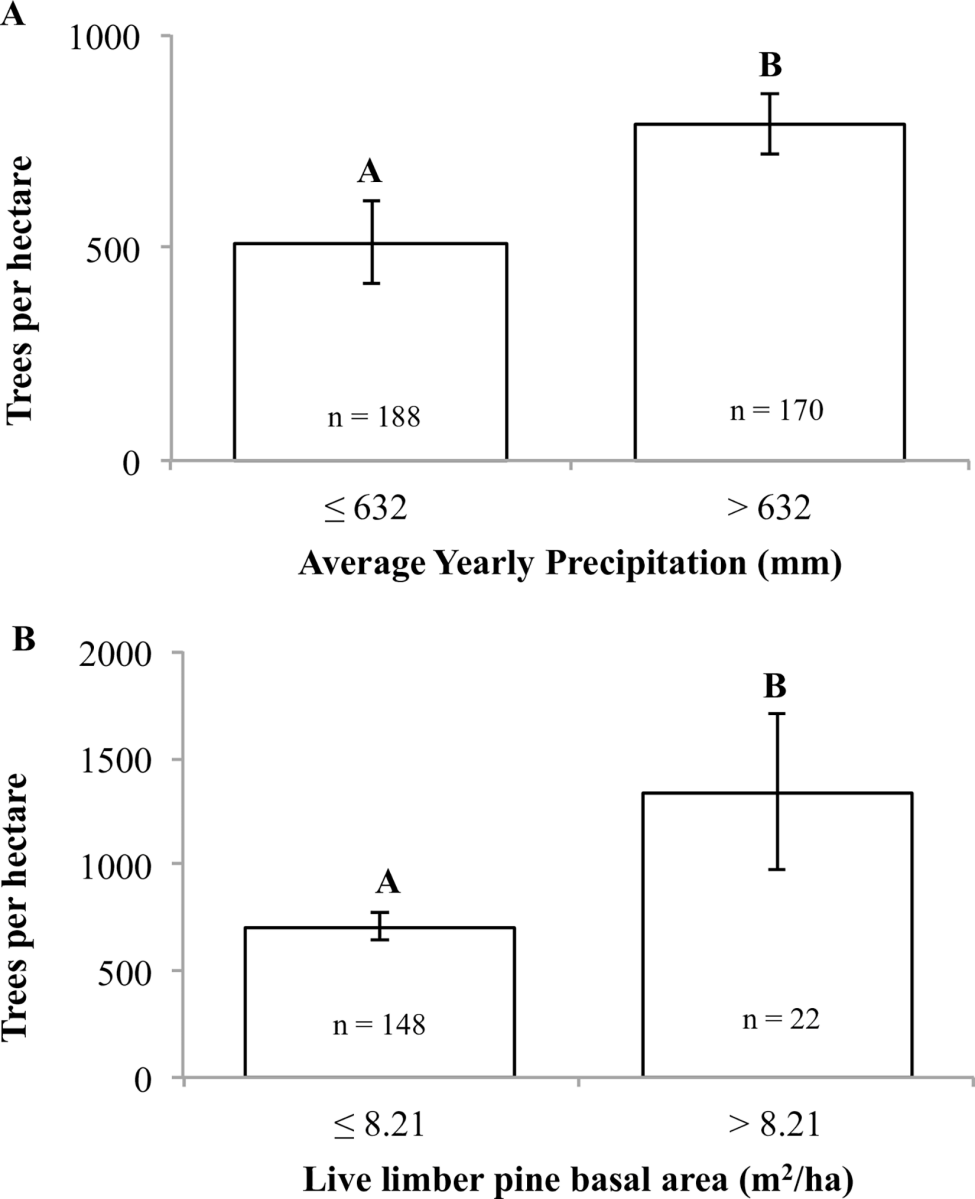
Average density of limber pine less than 2.54 cm in dbh across the Intermountain West. Using a conditional inference trees significant splits occurred based on average yearly precipitation and limber pine basal area per hectare. A) The first significant split (p<0.001) was on plots with greater than 632 mm and those less than or equal to 632 mm of average yearly precipitation. B) Of the 170 plots with greater than 632 mm of average yearly precipitation, a second significant split (p = 0.021) occurred based on limber pine basal area. Letters represent significant differences. Errors bars represent stand errors. N is the total number of plots in each of the bars. One plot with greater than 10,000 regenerating limber pine per hectare was excluded from this analysis.

By using a conditional inference tree, a more informative relationship was observed between limber pine regeneration and average annual precipitation and limber pine overstory basal area (Fig 5). Limber pine overstory basal area and yearly precipitation were important predictors of limber pine regeneration. On average, there was lower limber pine regeneration in plots with yearly precipitation under 632 mm (F_1,356_ = 19.4; p<0.001). Plots with less precipitation on average had 513 tph of limber pine compared to 792 tph on higher precipitation sites (Fig 5A). On sites with higher precipitation (> 632 mm), limber pine regeneration was greater when limber pine basal area was greater than 8.21 m^2^ha^-1^ (F_1,168_ = 5.3; p = 0.021). When limber pine basal area was lower, limber pine regeneration averaged 707 tph compared to 1336 tph under higher limber pine basal (Fig 5B).

## Discussion

Limber pine is commonly described as having a broad ecological amplitude. However, our study is one of the first to document general trends in limber pine stand dynamics across its broad geographic and elevational range. Limber pine dominant communities (>75% of the total basal area) can occur across all age classes and over a range of annual average temperatures and precipitation values. Overstory limber pine dominance was not strongly correlated with climatic variables or indices. For example, the Ombrothermic Index (OI), a measure of growing season available moisture, was not related to limber pine dominance neither was limber pine dominance restricted to drier sites that are present at lower and upper treeline (lower OI values) (Fig 2). Additionally, limber pine overstory dominance did not follow the expected bi-modal distribution suggestive of competitive exclusion from mid-elevations. Rather, it had a flat distribution and was a consistent component (14–29% of stand basal area) of forest communities across broad elevation classes (Fig 3). Other species did vary in their dominance across the elevation classes. These elevation classes broadly capture forest zones in the Intermountain West [4]. Limber pine was observed to occur in all of these forest zones and was a consistent component, interacting with species with widely different silvics [50]. While, plants do not respond directly to elevation, elevation is associated with important changes in temperature and precipitation in the mountainous regions [51–53].

Some limitations of our results include the lack of localized physiographic patterns or soil substrate and moisture holding capacity that greatly influences individual and stand growth. Additionally, the data used for this study only looked at plots that contained limber pine. Overstory dominance of all overstory species, including limber pine, would change if all FIAD plots were included in this analysis and would likely result in figures developed by Peet [4]. The use of the national FIAD reflects an unbiased estimate of the relative distribution of limber pine on the landscape [54]. Allowing our study to broadly characterize the relationship between limber pine dominance and environmental variables.

Our hypothesized description of limber pine as a poor competitor with a narrow realized niche highlights the lack of understanding of important aspects of stand dynamics for this species. The majority of plots with overstory limber pine received moderate (400–900 mm) to high (> 900 mm) average annual precipitation. Plots spanned a range of successional stages, forest conditions, and locations [55]. This was an observational study; density and environmental variables were not controlled or manipulated. Therefore, our results cannot elucidate the exact mechanisms or variables that define the realized niche of limber pine.

Contrary to our expectation of limber pine having a bi-modal distribution where it was competitively excluded under more mesic conditions, limber pine was observed to be generalist [14]-[16]. Limber pine’s distribution has many similarities to a well-studied generalist, aspen. Aspen also did not follow a uni-modal or bi-modal distribution but was a consistent component (~5% of stand basal area) across elevation classes where limber pine was observed. Across the Intermountain West, aspen occurs as a minor to major component of forest communities from lower to upper tree line, very similar to limber pine [56]. Aspen’s functional role, once thought to be essentially limited to that of a disturbance dependent, poor competitor, has been expanded and is thought to be more complex [57].

Our ecological understanding of limber pine is also currently expanding. This expansion suggests that limber pine may play more than one functional role (stress tolerator) in forest communities in the Intermountain West. Based on our observations, we propose three functional types for limber pine: dominant self-replacing; mixed-species; and invading. Limber pine dominant self-replacing communities generally have low diversity and lower density; this functional type is representative of the conventional view of limber pine communities. In mixed-species communities, limber pine adds species and structural diversity to a variety of forest communities across a wide range of environmental and stand conditions. This added diversity may increase forest resilience. Very little information is known about the role limber pine plays when it is a minor component. Finally, the invading limber functional type highlights the dispersal ability of limber pine; many plots with limber pine regeneration did not have live limber trees present in the overstory.

Limber pine seeds are dispersed by multiple species including the Clark’s nutcracker [58], [59]. Clark’s nutcracker can cache seeds more than 20 km from the source tree. Other common tree species of the montane and subalpine forest zones, e.g., lodgepole pine, Engelmann spruce, and ponderosa pine (*Pinus ponderosa* Dougl. ex Laws.), have very limited long distance seed dispersal; over 80% the seeds of these species fall within 75 m of the parent tree [60]. The Clark’s nutcracker prefers to cache seeds on windswept ridges and areas with early spring ground exposure but cache sites can occur in many different microenvironments [12], [58]. The mutualistic relationship between the bird and tree greatly influences the stand dynamics and structure. The timing and species of seeds arriving to a recently disturbed site has been shown to greatly influence composition [61]. Chance dispersals combined with limber pine’s broad ecological amplitude may be why we found low correlations between environmental variables or indices and limber pine overstory dominance.

However, we did observe a relationship between limber pine regeneration and limber pine basal area and average annual precipitation (Fig 5). Greater limber pine regeneration was associated with both greater average annual precipitation and greater limber pine basal area. Our results are consistent with other recent studies, which observed a negative relationship between moisture stress and limber pine regeneration [33], [62–64]. The link between moisture stress and decreased regeneration success relates to the fundamental ecology and the applied management of this species [65]. It also highlights important differences between the regeneration niche of seedlings and saplings and the realized niche of overstory limber pine. Shifts in environmental requirements during different life stages, especially for long-lived individuals like trees, are common [66], [67]. Environmental requirements are generally characterized based on mature, overstory individuals; however, during the seedling and sapling stage the species may be more sensitive to environmental conditions [68]. As climatic conditions continue to shift, it will be important to account for differing environmental requirements of seedlings, saplings, and overstory individuals to ensure continued forest cover.

An additional concern in western forests is insect outbreaks. Many forests types are experiencing large insect outbreaks including spruce beetle (*Dendroctonus rufipennis*) in spruce-fir forests, pinyon pine beetle (*Ips confuses*) in pinyon-juniper woodlands, Douglas-fir beetle (*Dendroctonus pseudotsugae*) in Douglas-fir forests, western spruce budworm (*Choristoneura occidentalis*) in numerous species commonly occurring in the montane and subalpine forest zones, and mountain pine beetle in pine forests [69]. For all of these insects except the mountain pine beetle, limber pine is not a host species and its presence may increase stand resistance and resilience to impacts from insect outbreaks [70]. In some systems, the presence of limber pine may limit the severity of a potential insect outbreak (associational resistance [71–73]) or in the event of an outbreak, maintain live forest cover and/or facilitate the re-establishment of forest cover after an outbreak (resilience).

Resistance and resilience of limber pine may be more limited in areas highly impacted by the invasive white pine blister rust (*Cronartium ribicola* J. C. Fisch. ex Rabenh.*)*. White pine blister rust has been shown to be one of many factors which increase selection by the mountain pine beetle in whitebark pine [74]. However, the Cr4 allele, associated with blister rust resistance, has been identified in some populations of limber pine [75]. Additional, research is needed on the complex interactions between forest health, disturbance dynamics, and climate change in limber pine and other five needle pines [76].

Limber pine, while often a minor component of overstory species composition, likely has a disproportionately large impact on forest communities in the Intermountain West. As both a minor and major component of different forest communities, limber pine may be extremely important as forest managers focus on increasing resistance and resilience to climate change [70], [77]. This study documented the basic forest dynamics and amplitude of limber pine across a broad geographic and elevation gradient. Limber pine was observed to occur from lower to upper treeline in a variety of different forest community types, supporting the description of limber pine as a generalist. This shift in our understanding of limber pine’s functional roles will be important for natural resource managers to incorporate into management planning. As forests are impacted by interactions between climate change and disturbance regimes, managers will be challenged to maintain resistance and/or increase resilience in these systems. The presence of a generalist like limber pine will offer managers flexibility in an uncertainty future.
